# Lack of sex- and gender-disaggregated data in diagnostics: findings from a scoping review of five tracer conditions

**DOI:** 10.3389/fpubh.2024.1484873

**Published:** 2025-01-28

**Authors:** Vishwanath Upadhyay, Rishabh Gangwar, Gabrielle Landry Chappuis, Mikashmi Kohli

**Affiliations:** ^1^Department of Molecular Medicine, Jamia Hamdard (Deemed to be University), New Delhi, India; ^2^Indian Council of Medical Research-National Institute of Malaria Research (NIMR), New Delhi, India; ^3^FIND, Geneva, Switzerland

**Keywords:** sex- and gender-disaggregated data in diagnostics, gender disparities in diagnosis of tuberculosis, coronavirus disease 2019 (COVID-19), diabetes, malaria, and schistosomiasis, sex- and gender-based analysis, sex- and age-disaggregated data

## Abstract

**Background:**

Sex and gender can affect all aspects of health-related behavior, yet there is limited information on how they influence diagnosis of any health condition. This scoping review examined the extent to which sex- and gender-disaggregated data on diagnostics are available for five tracer conditions: tuberculosis, coronavirus disease 2019 (COVID-19), diabetes, malaria, and schistosomiasis.

**Methods:**

Publications were searched between 2000 and 2022 on PubMed and Google Scholar and screened for relevance. Extracted data were analysed using descriptive quantitative and qualitative approaches.

**Results:**

We identified 29 relevant articles for tuberculosis, four for diabetes, six for schistosomiasis, eight for COVID-19, and three for malaria. For tuberculosis, most studies looked at gender-based barriers to diagnosis and disparities in health-seeking behaviors that predominantly affected women. For diabetes, studies noted that women had lower odds of being screened for prediabetes and potentially lower quality of care versus men. For schistosomiasis, studies suggested lower sensitivity diagnostic methods among women than men and low awareness of the disease. Studies suggest that women are less likely to be diagnosed for COVID-19 in certain settings. Studies on malaria reported that women show different health-seeking behaviors to men.

**Conclusion:**

This scoping review highlights a concerning lack of sex- and gender-disaggregated data on diagnostics. Consequently, further work is required to develop and implement an appropriate framework to assess gender and sex-related data around testing and diagnosis.

## Introduction

Diagnosis is a fundamental, essential component of healthcare systems. However, around half of the global population has little or no access to diagnostics, making diagnosis the single largest gap in the cascade of care (1). Access to diagnosis is also highly inequitable and people who are poor, less educated, and marginalized, including based on gender and sex, often face greater difficulties accessing diagnostics (1).

Gender can affect all aspects of health-related behaviors, as well as access to diagnosis (2). Gender can be defined as the socially constructed roles, behaviors, expressions, and identities of girls, boys, women, men, and gender-diverse people. Gender relations are hierarchical and unequal power relations between genders can impact health-related behaviors, including of health professionals, researchers, and clients or patients, impacting access to diagnosis and treatment (3). Gender can also produce inequalities that intersect with other socioeconomic inequalities (2). Because of these inequalities, women and girls often face greater barriers to accessing health care and information than men, as a result of lower decision-making power, restrictions on mobility, fewer financial resources, and discriminatory attitudes of healthcare providers (2). In contrast, sex refers to biological attributes, including physical features, chromosomes, gene expression, hormones and anatomy, which relate to male, female, or intersex attributes. Biological and physiological differences between sexes can produce differences in susceptibility to diseases, the progression of diseases, and treatment and health outcomes (4).

Consequently, ensuring equitable access to healthcare requires work to address gender- and sex-related barriers to care. This includes closing gaps around diagnostics, whether these occur as a result of sex-based differences in test performance or because of the different needs of sex/gender groups for diagnostic tools and services. However, the absence of robust sex-disaggregated data and gender analyses make it challenging to identify sex- or gender-based disparities and their causes and consequences across the cascade of care. In addition, sex and gender are often conflated, which complicates understanding of what factors underlie the observed differences and barriers to accessing diagnostics. The Sex and Gender Equity in Research (SAGER) and Guidelines for Accurate and Transparent Health Estimates Reporting (GATHER) guidelines (3, 5), as well as a recently published roadmap (6), are available to improve the collection of sex- and gender-disaggregated data. However, their application to research has been limited.

To address the critical sex and gender information gap, we undertook a scoping review to determine the extent to which sex-disaggregated data and gender analyses, are available for five tracer conditions: Tuberculosis (TB), coronavirus disease-2019 (COVID-19), diabetes, malaria, and schistosomiasis. These were selected to cover a spectrum of priority communicable and non-communicable diseases, as well as neglected tropical diseases.

## Methods

We conducted a scoping review using the JBI scoping review methodology (7) to evaluate sex and gender-disaggregated data for the above-mentioned tracer conditions with four main concepts, outlined in Table 1.

**Table 1:** Search strategy for scoping review by concept.

	Concept 1	Concept 2	Concept 3	Concept 4
Key concepts	Sex/gender disaggregated data and analysis on different stages of the diagnostic value chain for adults seeking care/diagnostics for the tracer conditions.	Adults’ health-seeking behaviors for the tracer conditions based on their sex/gender.	Compounding barriers to diagnostics based on sexual orientation, gender identity, and socioeconomic factors for adults (intersectional approach).	Gendered aspects of feasibility and acceptability of testing among adults seeking care/diagnostics of tracer conditions.
Synonyms, MeSH terms, free text, and natural language	Point-of-care diagnostic testing, molecular diagnostic techniques, general population	Treatment adherence and compliance, patient dropouts, patient participation, general population	Health services for transgender persons, sexual and gender minorities, health services accessibility	Patient acceptance of healthcare, healthcare acceptors, general population

### Identifying relevant studies

The study question was framed using the PICO criteria (Population/Problem, Intervention, Comparison, Outcome). For this study, the population was defined as people from low- and middle-income countries and high-income countries, the intervention was defined as care/diagnostics for the tracer conditions and outcome was defined as sex and/or gender-disaggregated data on various diagnostic value chain stages. The review did not include a comparator test. Using the Boolean operator, we combined PICO phrases with synonyms, MeSH terms, free text, and natural language to create a search strategy, summarized in Table 1. A protocol was developed and reviewed internally by FIND, but not published online.

### Search strategy

Publications were searched between 2000 and 2022 on PubMed and Google Scholar. The last search was conducted on 31 December 2022. Duplicates were found and eliminated when eligible articles were exported to an EndNote library. Publication titles were initially screened to assess if they were relevant for the objective of the scoping review. Abstracts were subsequently screened independently by two reviewers (VU, RG) who judged the relevance of publication based on the topic and tracer condition. Studies were excluded if they had no relevant comparison of patients by sex or gender, or if they were studies of only one sex or gender identity, were reviews and meta-analysis, were studies without the full text, or studies in any language other than English. Subsequently, the two reviewers (VU, RG) screened the full text of relevant articles to determine final inclusion.

### Collating, summarizing and reporting the results

Data were extracted for the key concepts, into a Microsoft Word table from full-text articles. Data were extracted by a single reviewer for one condition, and two reviewers for three conditions each (VU, RG). The extracted data were analysed using descriptive quantitative and qualitative approaches. Descriptive quantitative analyses included summarizing the number of articles, and whether the studies provided quantitative estimates on barriers to diagnostic access for the five investigated conditions. For qualitative data, we used the four primary concepts (summarized in Table 1) to recognize themes that fit best within each of these four concepts. As many of the studies referred to in this paper may conflate sex and gender when describing individuals, we applied the terms used in the studies themselves (e.g., male, female, women and men), or used terms in line with whether the study investigated sex or gender, if specified. Elsewhere we use the terms male, female, women, and men interchangeably (for example, when summarizing results from multiple studies that use both terms), although, we propose more specific use of terminology going forward.

## Results

**Figure 1:**
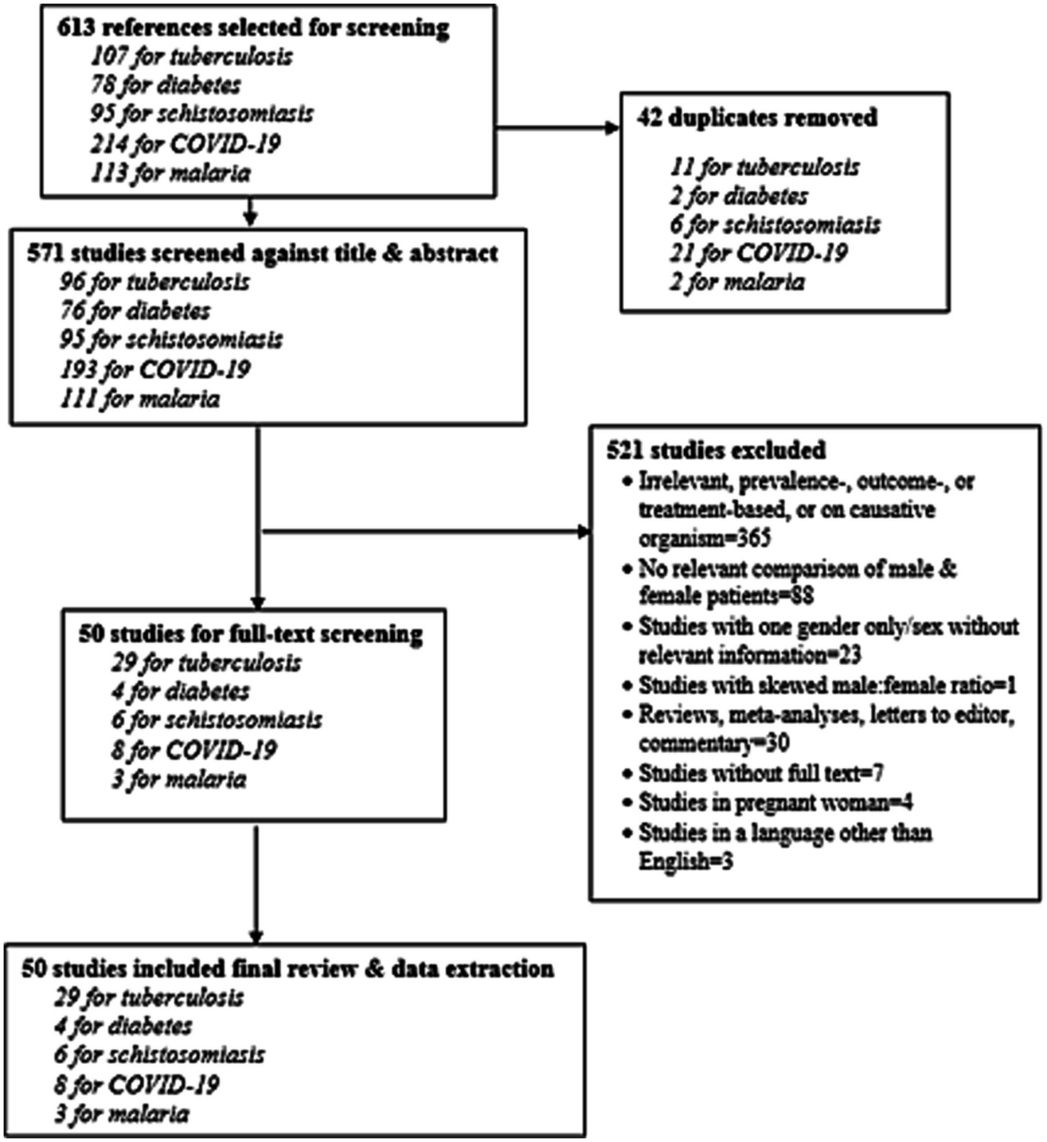
PRISMA diagram of evaluated and included studies.

Findings are presented by tracer condition. Our search strategy yielded 613 citations, of which 50 studies met the screening criteria (Figure 1).

### Gender/sex differences in tuberculosis diagnosis

Our search strategy yielded 107 citations, with 29 articles screened in full-text form and included in the review (Supplementary Figure S1) (8–36). Overall, the studies included in this review are both qualitative and quantitative, based on experimental and survey-based methods addressing different aspects of sex and gender-based disparities in the diagnosis of TB (Table 2).

**Table 2:** Overview of studies included for tuberculosis scoping review.

	Concepts	No. of studies
1	Sex/gender-disaggregated data and analysis on different stages of the diagnostic value chain for adults seeking care/diagnostics of TB (10, 13, 15, 25, 26, 28, 31, 33)	8
2	Adults’ health-seeking behaviors for TB based on sex/gender (9, 10, 16, 17, 19–22, 24, 26–31, 34, 36)	17
3	Compounding barriers to diagnostics based on sexual orientation, gender identity, and socioeconomic factors for adults (intersectional approach) (8, 9, 11, 12, 14–23, 25–32, 34–36)	25
4	Gendered aspects of feasibility and acceptability of testing among adults seeking care/diagnostics of TB (28, 29, 35)	3

#### Concept 1. Data on gender/sex differences in the diagnostic value chain stages for tuberculosis

Eight studies reported differences between the accuracy of diagnostic tests for TB among males and females (10, 13, 15, 25, 26, 28, 31, 33). Across the studies, the main causes of sex/gender differences in TB diagnosis were noted as the quality of specimens, use of less sensitive methods such as Ziehl-Neelsen for detecting acid-fast bacilli instead of molecular tests like Xpert *Mycobacterium tuberculosis* (MTB)/rifampicin (RIF) (10, 25, 26, 28, 31), and delay in specimen submission (15).

One study of individuals with suspected TB at an urban clinic in Nairobi, Kenya identified that females are more likely to produce suboptimal specimens (31). Other studies noted that there is anecdotal evidence that women are less likely to provide good-quality sputum, leading to a lower yield from smear microscopy (10).

A study from Myanmar reported sex-based differences in the diagnosis of rifampicin-sensitive and resistant TB using the Xpert MTB/RIF assay, with male patients more likely to test positive than females (47% vs. 39%) (33). Female patients also had a slightly higher proportion of rifampicin resistance (11·4%) than males (9·3%), although no reason was given for this difference (33).

Males are found to have a bias towards having sputum smear-positive pulmonary TB (13). Data from a large UK TB study were used to evaluate whether the immune response bias was important in terms of diagnosis or the ability to predict the onset of disease in persons exposed to TB. Females with sputum smear and culture-positive TB were found to have stronger tuberculin responses, whereas weak responses were seen in those who were screened for TB but did not develop the disease (13). Contrastingly, males had higher interferon-gamma responses than females to TB antigens ESAT-6 and CFP-10 (13).

#### Concept 2. Adults’ health-seeking behaviors for tuberculosis based on sex/gender

In Concept 2, 17 studies reported differences between healthcare-seeking behavior among male and female TB patients (9, 10, 16, 17, 19–22, 24, 26–31, 34, 36). Key causes noted as contributing to the differences between male and female healthcare-seeking behaviors included that women may seek healthcare much later than men resulting in delayed diagnosis of TB (20, 24, 29, 36) and that women are less likely to report cough and TB symptoms (28). In addition, healthcare-seeking behavior among women can be associated with social stigma (34). Another study from India reported that more females (58%) were scared than men (50%) while disclosing TB symptoms (30). A study from India also reported that more women (71%) than men (69%) utilized general healthcare and took self-treatment for TB symptoms (19). In Peru, Onifade et al. reported that women generally do not seek clinical investigations like X-rays and TB diagnostic tests due to a lack of money (29).

Furthermore, studies reported differences in where women seek care. Two studies noted that women are more likely to visit private clinics than men (16, 22). An assessment of health-seeking behavior in Indonesia found that women were more likely to seek care in private primary clinics compared with men, who opted for Level 0 pharmacy visits (16). A study conducted in Alexandria, Egypt, reported that more women than men had attended a private clinic for the diagnosis of TB (28·8% versus 14·7%) (22). Further, a study from China reported that women are more likely than men to visit the village clinic (49·1% vs. 44·6%, respectively) and local drug store (17% vs. 8·9%, respectively) (36).

#### Concept 3. Compounding barriers to diagnostics based on sexual orientation, gender identity, and socioeconomic factors

Gender/sex-based barriers to accessing diagnosis and care for TB were reported in 25 studies (8, 9, 11, 12, 14–23, 25–32, 34–36). Some studies noted that TB is associated with greater social stigma for women versus men (14, 17, 27, 34, 35). Females may also face barriers to accessing healthcare generally, because more women than men have limited finances, may be dependent on family members, and have a limited role in family decision-making (15, 29, 35). Household responsibilities and childcare can also lead females to receive delayed healthcare services (29, 35). Females may also have less awareness of TB than males, due to lower levels of education and access to information sources like television and radio (11, 17, 30).

However, one study of gender differences among TB patients in Guinea-Bissau found that although more women sought help for symptoms of pulmonary TB, more men were diagnosed. As women did not have more clinically severe diseases than men and did not drop out of diagnostic testing, the authors conclude that the gender gap is unlikely to be solely a result of differences in care-seeking behavior or diagnostic procedures in this setting (12).

In terms of specific populations, females from rural areas are more likely to receive delayed healthcare services (15), as are younger and divorced/separated women (23). Women may also be unable to produce sputum in front of male doctors or are less likely to be offered sputum test (35).

#### Concept 4. Gendered aspects of feasibility and acceptability of testing among adults seeking care/diagnostics of TB

Three studies reported implicit biases of healthcare providers based on sex or gender (28, 29, 35). A study of gender-related factors associated with delayed TB diagnosis in Eastern Europe and Central Asia identified that women in Georgia, Kazakhstan, and Tajikistan deprioritized seeking diagnosis for TB because of their lack of access to financial support, and obligations to care for family and young children (35). The study was also provided insights from gender-diverse people in Georgia, who reported stigmatization by health service providers in medical institutions (35). In Uganda, clinicians refer fewer female patients than males for acid-fast bacilli testing for the diagnosis of TB (28). In addition, the study in Peru identified that both patients and healthcare workers commonly believe that women’s health inherently has a lower priority than men’s health (29).

### Gender/sex differences in diabetes diagnosis

Our search strategy yielded 78 citations (Supplementary Figure S2). Of these, four articles met the inclusion criteria (37–40).

#### Concept 3. Compounding barriers to diagnostics based on sexual orientation, gender identity, and socioeconomic factors

A mixed-methods assessment in the US found that men had 19% higher odds (95% CI:1·09–1·30) of being screened for prediabetes than women, possibly due to the higher prevalence of hypertension (a consideration for screening) among men (37).

A cross-sectional study in Germany identified that quality of care for patients with type 2 diabetes is influenced by the gender of the physician (39). Women physicians were found to provide an overall better quality of care, especially in prognostically important risk management (39).

#### Concept 4. Gendered aspects of feasibility and acceptability of testing among adults seeking care/diagnostics

A study of sex-related psychological differences in type 2 diabetes mellitus found that females reported lower satisfaction with social support. However, females on oral hypoglycaemic drugs were better informed about type 2 diabetes than males (38). Gender differences were also found across various quality-of-care measures for diabetes. Both men and women with diabetes were found to have quality-of-care deficits. Still, women generally reported lower quality of care than men among most measures for cardiovascular disease and diabetes.

### Gender/sex differences in schistosomiasis diagnosis

The search identified 101 citations, of which 6 met the inclusion criteria (Supplementary Figure S3; Table 3).

**Table 3:** Overview of studies included for schistosomiasis scoping review.

	Concepts	No. of studies
1	Sex/gender-disaggregated data and analysis on different stages of the diagnostic value chain for adults seeking care/diagnostics of schistosomiasis (41, 42)	2
2	Adults’ health-seeking behaviors for schistosomiasis based on sex/gender (43)	1
3	Compounding barriers to diagnostics based on sexual orientation, gender identity, and socioeconomic factors for adults (intersectional approach) (43–46)	4
4	Gendered aspects of feasibility and acceptability of testing among adults seeking care/diagnostics of schistosomiasis (43, 44)	2

#### Concept 1. Data on gender/sex differences in the diagnostic value chain stages for schistosomiasis

A retrospective analysis of 1700 participants across eight studies conducted in Tanzania between 2010 and 2016 found decreased sensitivity of egg microscopy in women (41). In HIV-noninfected women and men, egg microscopy had a sensitivity of 38 and 62%, respectively (38% versus 62% difference, *p* < 0·001) and in HIV- for *Schistosoma* infection (41). This finding was maintained, although smaller, among HIV-infected individuals (egg microscopy sensitivity of 33% in women versus 41% in men, *p* = 0·23) (41).

A study analysing f self-reported urinary schistosomiasis in school children from Tanzania found that the specificity of self-reported schistosomiasis was 100% in older girls, indicating correct self-diagnosis by all uninfected girls (42). However, the sensitivity of self-diagnosis among girls was lower, with results showing incorrect self-diagnosis more often by infected girls than infected boys. In addition, infected older girls were more likely to report their infection status incorrectly than infected younger girls (42).

#### Concept 2. Adults’ health-seeking behaviors for schistosomiasis based on sex/gender

One article reported work to train healthcare workers on how to improve the prevention, diagnosis, and treatment of female genital schistosomiasis (FGS) (43). Virtual workshops were held to establish standardized skills across the continuum of care for FGS, including for prevention diagnosis and management (43).

#### Concept 3. Compounding barriers to diagnostics based on sexual orientation, gender identity, and socioeconomic factors

A study of schistosomiasis-related perceptions, attitudes, and treatment-seeking practices in Tanzania identified social stigma associated with various manifestations of schistosomiasis (44). Boys, girls, and adults were noted as being shy to tell parents and doctors of their symptoms because of shame, fear of disclosure to others, and the association with promiscuity (44). Another study highlighted low awareness of the disease in Sub-Saharan African communities and health systems, which acts as a barrier for women in terms of addressing the symptoms and complications associated with FGS (43).

A qualitative assessment of knowledge gaps around FGS among communities in endemic districts of Zanzibar and mainland Tanzania identified that most participants, male and female, lacked knowledge of FGS (45). Most participants also reported a lack of knowledge that urogenital schistosomiasis can affect female reproductive system (45). Adolescent girls and women with symptoms of FGS were reportedly stigmatized and perceived as having a sexually transmitted disease (STD) (45).

Another study conducted an epidemiological and socioeconomic analysis of FGS across eleven rural fishing communities in Cameroon (46). The study identified that inadequate diagnostics and treatments for FGS are major causes of suffering among women and girls with the condition, and instead often mistreated for STDs and other infections (46).

#### Concept 4. Gendered aspects of feasibility and acceptability of testing among adults seeking care/diagnostics

In Tanzania, healthcare professionals stated that their services, whether they were focused on modern or traditional medicine, were complementary to one another (44). Traditional healers mostly with abdominal and infertility problems among women (44).

Another study highlighted the common cycle of FGS misdiagnosis and treatment, where women with symptoms are presumptively treated for STDs (43). When the woman’s symptoms do not resolve, healthcare workers may assume non-compliance or re-infection and the woman will be asked to repeat treatment (43). Eventually, the woman may be referred to the next-level health centre, where she may receive additional examinations, but will likely receive next-line treatment for STDs (43). Each step of the cycle results in financial and opportunity costs for women, and more time spent with untreated FGS and its complications (43).

### Gender/sex differences in COVID-19 diagnosis

A total of 214 citations were identified from the search strategy (Supplementary Figure S4). Of these, eight studies met the inclusion criteria.

#### Concept 2. Adults’ health-seeking behaviors for COVID-19 based on sex/gender

Countries, in which females face more discrimination within families and have limited access to resources, education, and finance, report greater gaps between male and female rates of COVID-19 positive cases and mortalities (47, 48). A retrospective study of COVID-19 sex-disaggregated data from 133 countries found that although most countries tend to report approximately equal COVID-19 infection and death rates for men and women, certain countries reported far higher case rates among men. For example, the proportion of confirmed COVID-19 cases that are men is 88% in Bahrain, 85% in Qatar, 75% in Saudi Arabia and South Sudan, 74% in Pakistan, and 71% in Bangladesh (47). However, some countries report a slightly higher proportion of COVID-19 cases among women, including Ukraine, Moldova, Poland, Latvia, Jamaica, Georgia, and Armenia (47).

Another analysis of sex-segregated COVID-19 data for 72 countries suggests that countries with institutionalized gender disparities and poor healthcare access and quality tend to have higher male-to-female (M: F) ratios of confirmed COVID-19 cases. In the study, Cambodia had the highest M: F ratio of confirmed COVID-19 cases (4.08:1), followed by Pakistan (M: F = 2·85:1) and Nepal (M: F = 2·69:1) (48). The study also identified a positive correlation between the gender inequality index and M: F ratio (Spearman’s rho = 0·681, *p* < 0·001) and a negative correlation between healthcare access and quality index and M: F ratio (Spearman’s rho = −0·676, *p* < 0·001) (48).

#### Concept 3. Compounding barriers to diagnostics based on sexual orientation, gender identity, and socioeconomic factors

A study using the Dutch Lifelines COVID-19 Cohort (*N* = 74,722; 60·8% female) found no sex-related disparities in COVID-19 testing and diagnosis in the general population. However, among healthcare workers, females were less often diagnosed and tested than males (odds ratio = 0·54; 95% CI, 0·32–0·92 and odds ratio = 0·53; 95% CI, 0·29–0·97, respectively) (49).

People who identify as lesbian, gay, bisexual, transgender, or queer and questioning (LGBTQ+) confront numerous health inequities, which may increase their vulnerability to COVID-19 compared with the general population. In a nationwide survey of COVID-19 testing in LGBTQ+ populations in the United States, of the 1,090 participants, 182 (16·7%) had received a PCR test, with 16 (8·8%) receiving a positive result (50). Across the surveyed population, antibody testing rates were greater among gay cisgender men (17·2%) versus other sexual orientation and gender identity groups, non-US-born (25·4%) versus US-born participants, employed (12·6%) versus unemployed participants, and Northeast (20·0%) participants versus other regions (50). For PCR testing, positive PCR results were highest among cisgender gay males (16·1%) among sexual orientation and gender identity groups with sufficient numbers for comparison (50). The authors note that the disparities in testing and positivity, notably among gay males in this sample, highlight the importance of developing COVID-19 public health messages and programming for the LGBTQ+ population (50).

In a national online survey of US adult individuals belonging to sexual and gender minority groups, transgender and bisexual/pansexual people reported being more interested in a COVID-19 vaccine and an at-home test than cisgender and gay/lesbian respondents (51). Respondents who endorsed having intersex traits and people living with HIV were both less likely to be interested in an at-home COVID-19 test than those who did not endorse intersex traits or people living without HIV, respectively (51). These findings highlight critical differences in COVID-19 symptomology and prevention behaviors among sexual and gender minority groups, which should be considered when designing effective COVID-19 interventions (51).

A study of the prevalence and correlates of COVID-19 testing in an online sample of transgender and non-binary people (*n* = 536) discovered that certain identities were significantly more likely to test for severe acute respiratory syndrome coronavirus 2 (SARS-CoV-2) (52). Transgender and non-binary individuals from upper socioeconomic income backgrounds and Europe who reported active alcohol use disorder, limited access to gender-affirming surgery, income reduction of more than 20%, and mistreatment in a health facility due to gender identity had significantly greater probabilities of COVID-19 testing (all *p* < 0.05) (52).

A population-wide cohort study of all residents of Ontario, Canada, who received a nasopharyngeal swab for SARS-CoV-2 between 23 January 2020 and 26 May 2020 looked at sex- and age-specific differences in COVID-19 testing, cases, and outcomes (53). Except two age groups (ages 0–9 years and 70–79 years), males received less COVID-19 testing than would be expected for their age-based representation in the Ontario population (53). For those with COVID-19 infection, males also had higher rates of hospitalization (15·6% vs. 10·4%), intensive care unit (ICU) admission (4·1% vs. 1·7%), and death (8·7% vs. 7·6%) than females; these findings were consistent even when adjusting for age (53).

A cross-sectional analysis of data from a COVID-19 Surveillance and Outcomes Registry from the US reported that males were significantly more likely to test positive for COVID-19 (17.0% in males vs. 14.6% in females, odds ratio 1:20) (54). Irrespective of age, among hospitalised patients, males were more likely than females to experience medical complications, need ICU admission and mechanical ventilation, and have higher death rates (54).

### Gender/sex differences in malaria diagnosis

The search identified 113 citations, with three meeting the inclusion criteria (Supplementary Figure S5).

#### Concept 3. Compounding barriers to diagnostics based on sexual orientation, gender identity, and socioeconomic factors

A study of the factors affecting household choice of malaria treatment options in Ghana found that women from Hohoe (a rural area) were more likely than men to seek treatment for malaria from a public health care provider (55). Whereas, a study in Vietnam reported that the delay between first symptoms and seeking healthcare was significantly longer among female patients (median 3 days) compared with male patients (median 2 days; *p* < 0·001) (56).

In Uganda, a study of gender differences in the incidence of malaria at 12 public malaria reference facilities found that women were significantly more likely than men to report fever in the previous two weeks and seek care at local health centres (7·5% vs. 4·7%, *p* = 0·001) (57). However, female gender was also associated with a higher incidence of visits where malaria was not suspected (IRR = 1·77, 95% CI 1·71–1·83, *p* < 0.001) (57).

## Discussion

Overall, this scoping review identified a paucity of information on how sex and gender affect diagnosis. Across the five tracer conditions, the most information was available for TB, with 29 studies identified with relevant information. Overall, most studies on TB touch upon gender-based barriers to diagnosis and disparities in health-seeking behaviors, which predominantly affect women. Although eight studies looked at differences in diagnostic test performance for TB by sex/gender, these studies are relatively old and relate to older TB diagnostic techniques such as Ziehl-Neelsen testing, which have since been replaced with more sensitive fluorescent microscopy and nucleic acid amplification tests (e.g., GeneXpert testing). Consequently, further research is needed to understand if there are gender and sex differences in the performance of current standard-of-care testing methods for TB.

For diabetes, there was a concerning lack of data on gender- and sex-based inequalities in diabetes diagnosis, particularly given the growing prevalence of the condition, which now affects around 422 million people worldwide (58). Of the four identified studies, one in the US indicated that men have 19% higher odds of being screened for prediabetes than women, possibly due to a higher prevalence of hypertension among men (37). Two other studies suggest that women may experience lower quality of care and less social support than men for diabetes (38, 40).

For schistosomiasis, there was a similar lack of information on gender and sex differences around diagnosis, despite over 250 million people requiring preventive treatment for the condition in 2021 (59). Across the six studies on the topic, two suggest lower sensitivity diagnostic methods (egg microscopy and self-reported diagnosis, respectively) among women than among men (41, 42). Other studies report low awareness of the disease among men and women (43). Given the serious effects of FGS for women and difficulties in differential diagnosis of the condition, further research would be valuable to inform diagnostic approaches that are gender- and sex-responsive.

Only eight studies were identified on sex- and gender differences in COVID-19 diagnosis. The available studies suggest that women are less likely to be diagnosed with COVID-19 in lower-income countries and in countries where women face more discrimination and greater socioeconomic barriers than men (47, 48). However, a study of COVID-19 testing, cases, and outcomes in Canada found that men were less likely to be tested for COVID-19, yet had higher rates of COVID-19 infection and worse outcomes compared with women (53). Other studies found different rates of COVID-19 testing among different identities within the LGBTQ+ community, suggesting a need for targeted messaging for identities that were less likely to access testing. Together, the available studies suggest nuanced sex and gender differences in COVID-19 testing but the available data are too limited to draw firm conclusions at present.

For malaria, there was a notable lack of information about gender and sex differences in diagnosis, with only three studies identified. The studies, from Ghana, Viet Nam and Uganda, report that women show different health-seeking behaviors to men, with women more likely to seek treatment from a public health provider, experiencing a longer delay before seeking care, and reporting fever than men (55–57). Additional studies confirming whether these differences are common across different geographical areas would be useful to understand persistent sex and gender differences and barriers around malaria diagnosis.

Although the scoping review was comprehensive in its approach, our findings are limited by the paucity of evidence, particularly for certain conditions like malaria. In addition, we identified a lack of standardization in how sex and gender terminology is used (e.g., as seen in the interchangeable use of “women” and “females” in reports, regardless of whether sex or gender was investigated). Avoiding conflation of these terms moving forward will be a key part of improving the reporting of sex and gender differences in scientific studies.

In summary, this scoping review provides an initial comprehensive assessment of available information on sex and gender differences in diagnosis across five key tracer conditions. The findings highlight a concerning lack of sex- and gender-disaggregated data around diagnosis and access to diagnostics. This makes it challenging to develop evidence-based solutions to sex and gender disparities around diagnostics and ensure that everyone, regardless of their sex and gender, has equitable access to diagnosis and high-performing diagnostic tools and services. Consequently, further work is required, as a priority, to develop and use robust indicators and an appropriate framework to assess gender and sex-related data on diagnostics. At FIND, the learnings from this scoping review will be used to inform a sex/gender framework for diagnostic R&D and access. Insights will also be used to advocate for better data and analysis to improve diagnosis for everyone, regardless of gender and sex.

## Data Availability

The original contributions presented in the study are included in the article/Supplementary material, further inquiries can be directed to the corresponding author.
