# Supplementary material for: Lack of sex- and gender-disaggregated data in diagnostics: findings from a scoping review of five tracer conditions
Source: Front Public Health. 2025 Jan 28;12:1484873. doi: 10.3389/fpubh.2024.1484873 (PMC11810905; doi:10.3389/fpubh.2024.1484873)

Supplementary materials

Supplementary Figure 1. PRISMA diagram of evaluated and included studies for sex/gender differences in tuberculosis diagnosis

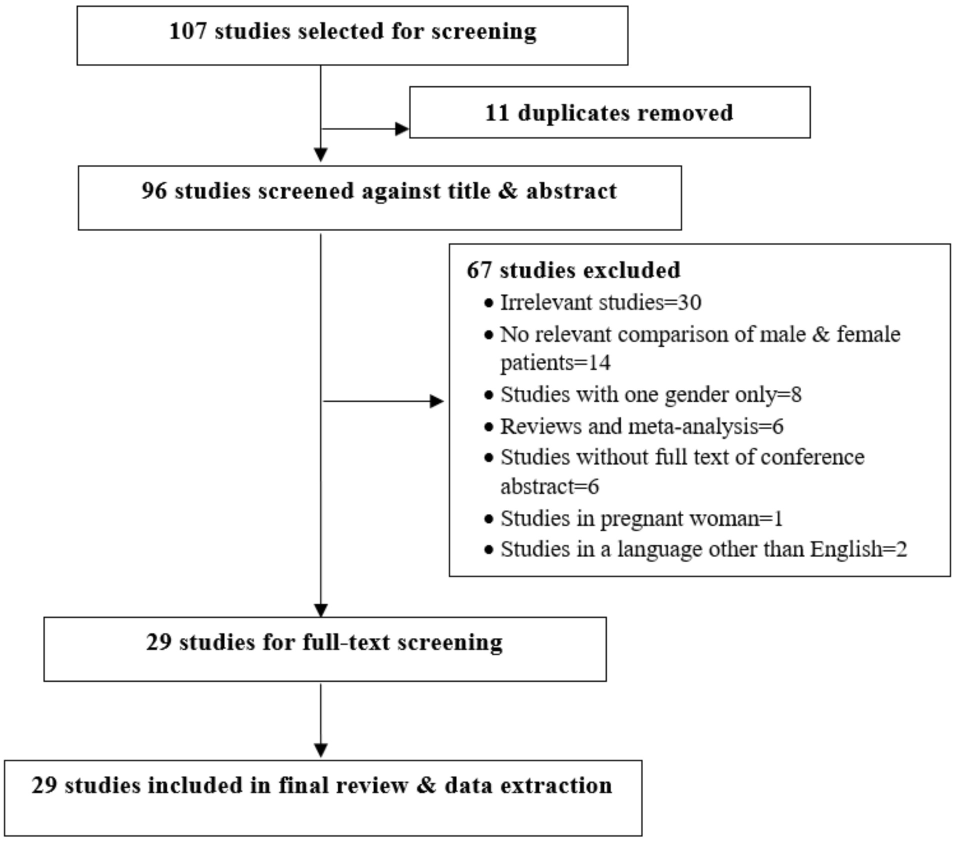

Supplementary Figure 2. PRISMA diagram of evaluated and included studies for sex/gender differences in diabetes diagnosis

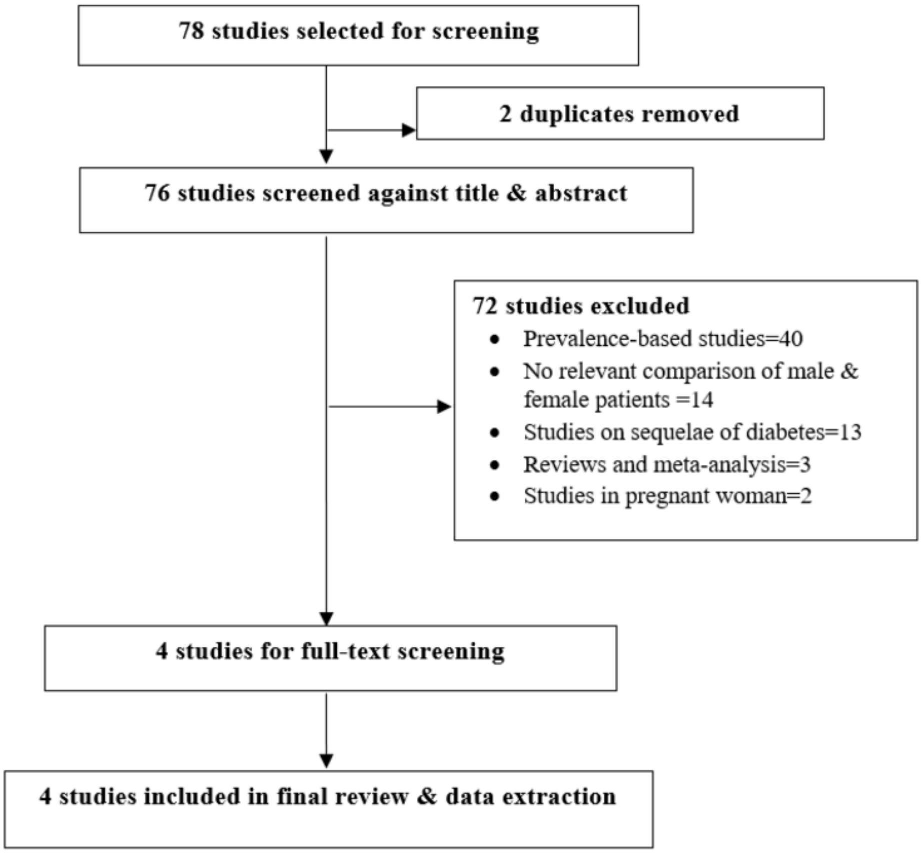

Supplementary Figure 3. PRISMA diagram of evaluated and included studies for sex/gender differences in diagnosis of schistosomiasis

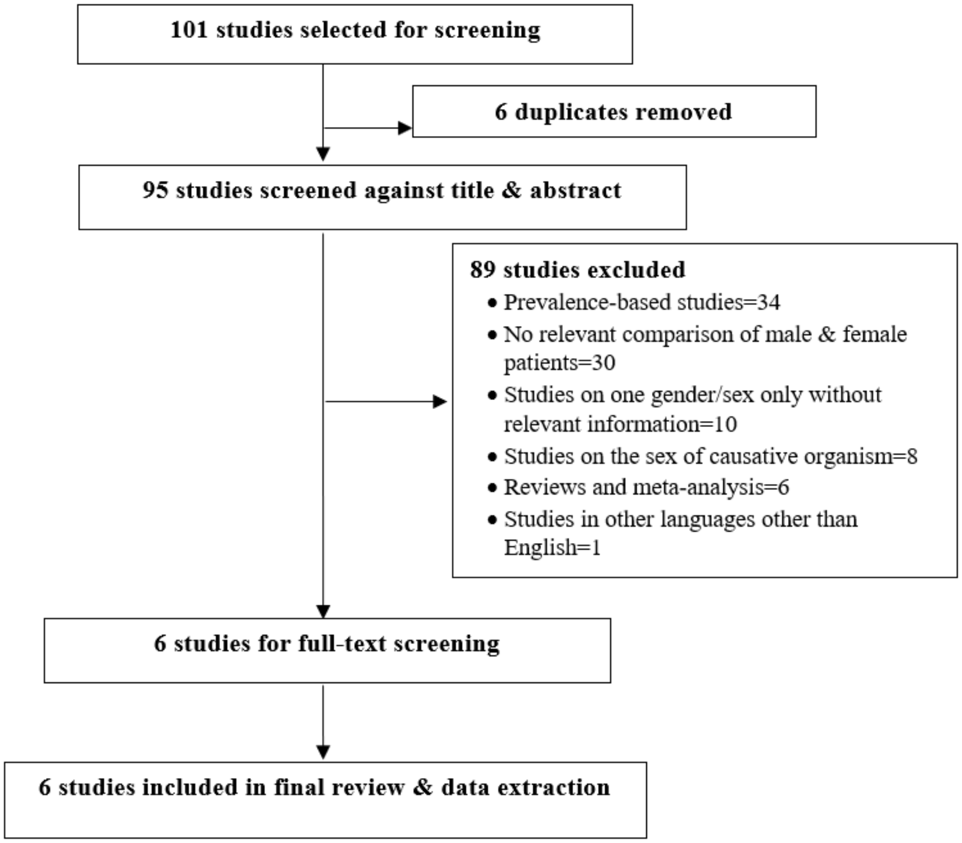

Supplementary Figure 4. PRISMA diagram of evaluated and included studies for sex/gender differences in COVID-19 diagnosis

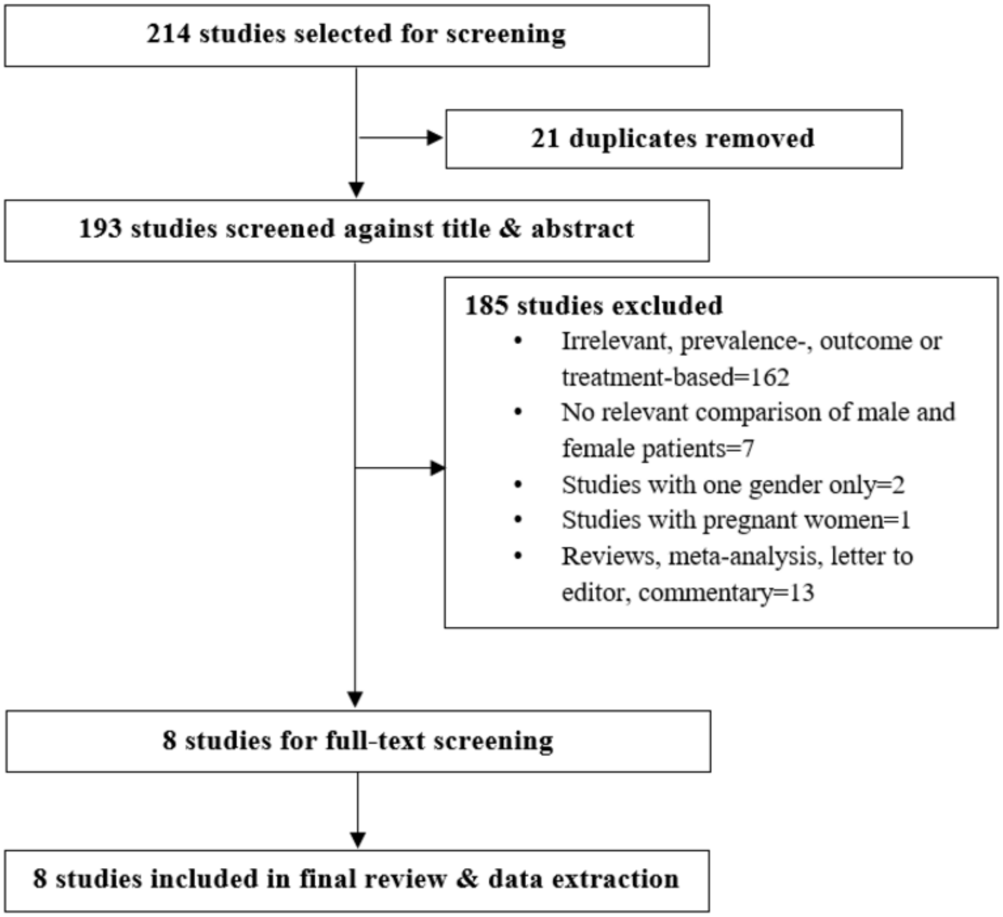

Supplementary Figure 5. PRISMA diagram of evaluated and included studies for sex/gender differences in malaria diagnosis

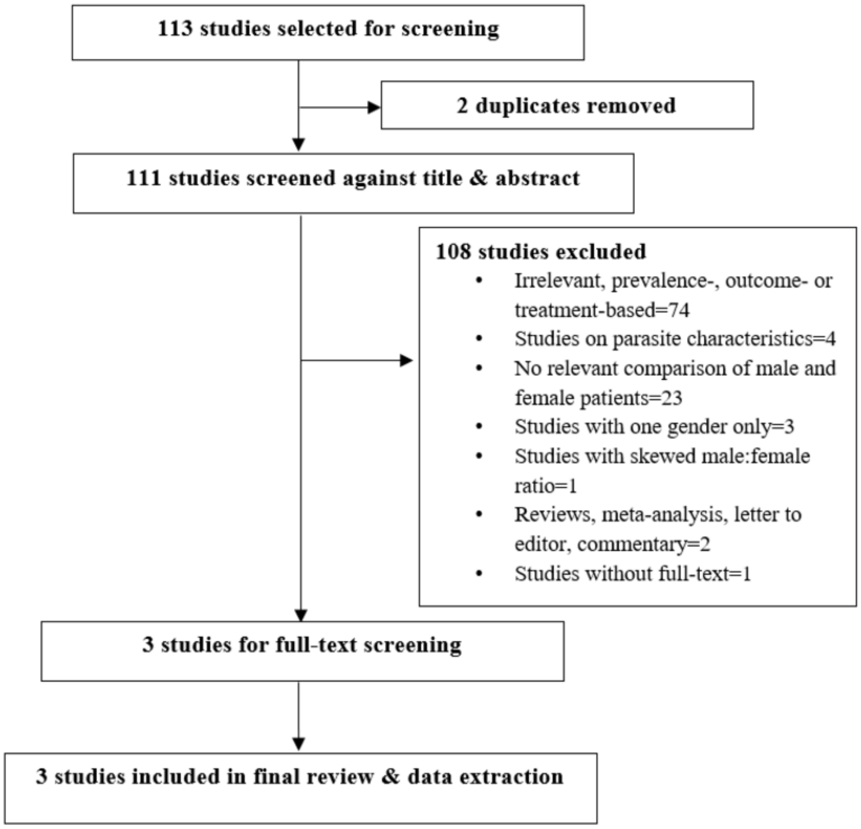

Supplement: Supplementary file 1 [file Data_Sheet_1.PDF]
